# Adrenomedullin and tumour angiogenesis

**DOI:** 10.1038/sj.bjc.6602832

**Published:** 2005-10-25

**Authors:** L L Nikitenko, S B Fox, S Kehoe, M C P Rees, R Bicknell

**Affiliations:** 1 Nuffield Department of Obstetrics and Gynaecology, The University of Oxford, John Radcliffe Hospital, Oxford, OX3 9DU, United Kingdom; 2 Nuffield Department of Clinical Laboratory Sciences, The University of Oxford, John Radcliffe Hospital, Oxford, OX3 9DU, United Kingdom; 3 Molecular Angiogenesis Laboratory, Cancer Research UK, Weatherall Institute of Molecular Medicine, The University of Oxford, John Radcliffe Hospital, Oxford, OX3 9DU, United Kingdom; 4 Institute for Biomedical Research, Birmingham University Medical School, Edgbaston, Birmingham B15 2TT, United Kingdom

**Keywords:** adrenomedullin, CRLR, CL, angiogenesis, tumour, endothelial cell

## Abstract

The angiogenic activity of peptide adrenomedullin (AM) was first shown in 1998 . Since then, a number of reports have confirmed the ability of AM to induce the growth and migration of isolated vascular endothelial and smooth muscle cells *in vitro* and to promote angiogenesis in xenografted tumours *in vivo.* In addition, knockout murine models point to an essential role for AM in embryonic vasculogenesis and ischaemic revascularisation. AM expression is upregulated by hypoxia (a typical feature of solid tumours) and a potential role as a regulator of carcinogenesis and tumour progression has been proposed based on studies *in vitro* and in animal models. Nevertheless, translational research on AM, and in particular, confirmation of its importance in the vascularisation of human tumours has lagged behind. In this commentary, we review current progress and potential directions for future research into the role of AM in tumour angiogenesis.

The increased risk of endometrial polyps, hyperplasia and cancer in women receiving tamoxifen treatment for breast cancer prompted our group to look for genes induced in endometrial isolates by tamoxifen, but not oestrogen. PCR differential display identified the vasoactive peptide adrenomedullin (AM) as one such gene (Zhao *et al*, 1998). We subsequently showed AM to be an endothelial cell growth factor, having potent *in vivo* angiogenic activity and promoting tumour growth in animal models (Zhao *et al*, 1998; Nikitenko *et al*, 2000; Oehler *et al*, 2001, 2002). The last 7 years have seen increasing interest in elucidating a role of AM in tumorigenesis. The purpose of this commentary is to review those studies and to identify current unanswered questions.

## ADRENOMEDULLIN

### Adrenomedullin peptide

Andrenomedullin is a 52-amino-acid peptide originally isolated from a human phaeochromocytoma (Kitamura *et al*, 1993a). It is produced through cleavage of a 185-amino-acid prohormone (pre-proadrenomedullin), which also yields proadrenomedullin *N*-terminal 20 peptide (PAMP). Andrenomedullin belongs to the calcitonin gene peptide superfamily based on its homology with calcitonin gene-related peptide (CGRP) and amylin (Poyner *et al*, 2002). The pleiotropic activities of AM were recognised within a few years of its initial characterisation as a vasodilatory peptide (Kitamura *et al*, 1993a; reviewed by Hinson *et al*, 2000). The expression and secretion of AM has been demonstrated in many tumours and biological fluids and it has been implicated in the modulation of numerous physiological processes (reviewed in Hinson *et al*, 2000). For example: (1) it is a *growth factor* (Hinson *et al*, 2000), (2) it is *angiogenic* in *in vitro* models and *in vivo*, (Zhao *et al*, 1998; Nikitenko *et al*, 2000; Kim *et al*, 2003), (3) it *inhibits apoptosis* in endothelium and isolated tumour cells (Kato *et al*, 1997; Martinez *et al*, 2002; Oehler *et al*, 2002), (4) it is a potent *vasodilator* (reviewed by Hinson *et al*, 2000), (5) it *regulates endothelial permeability* (Hippenstiel *et al*, 2002), and (6) it contributes to *adhesion and differentiation* of bone marrow-derived mononuclear cells into endothelial progenitor cells (Iwase *et al*, 2005).

### Regulation of adrenomedullin expression

Andrenomedullin expression is stimulated by cytokines and hypoxia and its promoter contains several putative hypoxia response elements (reviewed by Zudaire *et al*, 2003). Hypoxia induces AM expression and secretion in many cell types including carcinoma cell lines (endometrial, breast, neuroblastoma, colorectal) (reviewed by Zudaire *et al*, 2003) and endothelial cells (Nakayama *et al*, 1999; Nikitenko *et al*, 2003). Hypoxia is a frequent feature of the microenvironment in solid tumours and constitutes one of the driving forces of cancer growth and progression (Harris, 2002), and a role for AM as a promoter of these processes has been suggested (Oehler *et al*, 2001; Martinez *et al*, 2002; Ouafik *et al*, 2002).

### Biodegradation of adrenomedullin and the role of adrenomedullin binding protein

A specific AM binding protein (i.e., AMBP-1) has been described and characterised as human complement factor H (reviewed by Zudaire *et al*, 2003). Binding proteins can limit the transport of a peptide to the interstitial space and access to its specific receptors, modulate its biological activity, as well as protecting it from metabolic clearance by proteases and thereby prolonging the half-life in the circulation. This could explain why the growth promoting activity of AM is augmented in the presence of AMBP-1 (reviewed by Zudaire *et al*, 2003).

### Adrenomedullin receptors

**Figure 1:**
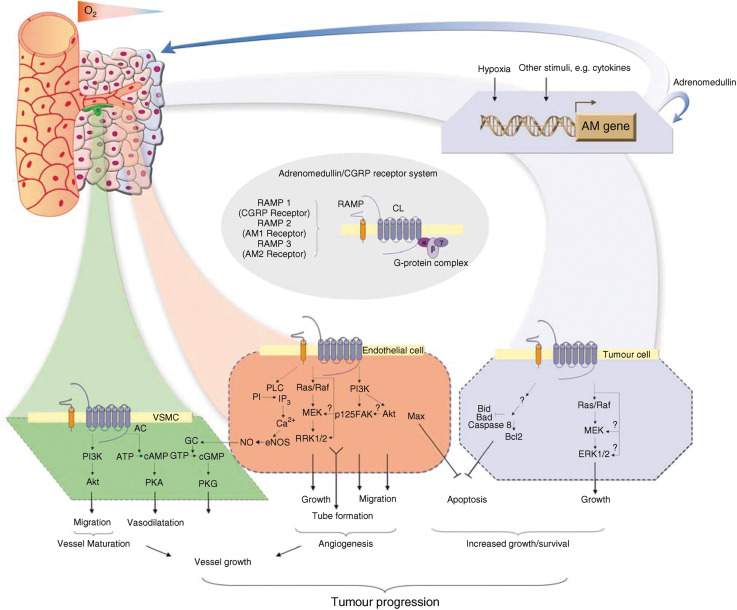
Role of adrenomedullin in tumour progression. The role of hypoxia and inflammatory cytokines in regulation of AM expression and secretion by tumour cells *in vivo* has been suggested. Adrenomedullin promotes formation of xenografted tumours by stimulation of autocrine growth and survival of tumour cells, and through paracrine effects on surrounding vessels. Possible intracellular signalling mechanisms underlying effects of AM in tumour microenvironment (in endothelial, vascular smooth muscle (VSMC) and tumour cells) suggest its potential role in tumorigenesis, resistance to chemotherapy and tumour progression. Based on McLatchie *et al* (1998), Shichiri *et al* (1999), Hinson *et al* (2000), Oehler *et al* (2001), Martinez *et al* (2002), Poyner *et al* (2002), Kim *et al* (2003) and Iwase *et al* (2005). AC=adenylate cyclase; GC=guanylate cyclase; PKA=protein kinase A, PKG=protein kinase G, PLC=phospholipase C, MEK=mitogen-activated protein kinase kinase; ERK=extracellular signal-regulated kinase (also termed MAPK).

AM mediates its activities via heterodimeric receptors that are composed of a seven transmembrane (7TM) G-protein-coupled receptor (GPCR) calcitonin-receptor-like receptor (CRLR, now known as CL; Poyner *et al*, 2002), and a receptor activity modifying protein (RAMP) (McLatchie *et al*, 1998). The RAMP family comprises three members (RAMP1, RAMP2 and RAMP3) that share less than 30% sequence identity but a common topological organisation. They are small intrinsic membrane proteins with a large extracellular *N*-terminus (∼100 amino acids), a single transmembrane domain, and a short intracellular domain (10 amino acids). Formation of heterodimers between RAMPs and CL is essential for proper cell surface targeting and to define ligand-binding selectivity of this GPCR (McLatchie *et al*, 1998). RAMP2 and RAMP3 promote the expression of AM receptors (termed AM1 and AM2, respectively), while coexpression of RAMP1 with CL leads to the formation of CGRP receptor (McLatchie *et al*, 1998; Poyner *et al*, 2002) (Figure 1). However, the mechanisms regulating CL and RAMPs expression remain poorly characterised.

### Adrenomedullin and cell signalling

Adrenomedullin signal transduction differs between cell types, involving several pathways (Figure 1). Increasing evidence has shown that activation or disruption of AM signalling may contribute to pathologies including ischaemia-induced damage and neoplastic growth (reviewed in Hinson *et al*, 2000; reviewed in Nikitenko *et al*, 2002). It follows that the development of drugs modulating AM activity hold the potential for pro- or antiangiogenic therapies.

## ADRENOMEDULLIN AND CANCER

Adrenomedullin has been shown to be involved in carcinogenesis and tumour progression by promoting tumour proliferation, angiogenesis and the inhibition of apoptosis. It is thought that inflammatory cytokines- and hypoxia–induced expression of AM by tumour cells drives these processes (Figure 1).

### Adrenomedullin and tumour cell proliferation

Several AM-overexpressing human carcinoma cell lines exhibit enhanced growth *in vitro* and *in vivo* to a varying degree (Oehler *et al*, 2001; Martinez *et al*, 2002). Thus, the endometrial cancer cell line RL95.2 overexpressing AM showed a marked growth increase, but no increase was seen with Ishikawa-transfected endometrial carcinoma cells (Oehler *et al*, 2001). Adrenomedullin also maintains cell proliferation of breast tumour cell lines T47D in serum-free conditions (Martinez *et al*, 2002). Finally, an anti-AM antibody significantly decreased *in vitro* and *in vivo* growth of U87 glioblastoma cells that have a high level of endogenous AM (Ouafik *et al*, 2002).

### Adrenomedullin and angiogenesis

**Figure 2:**
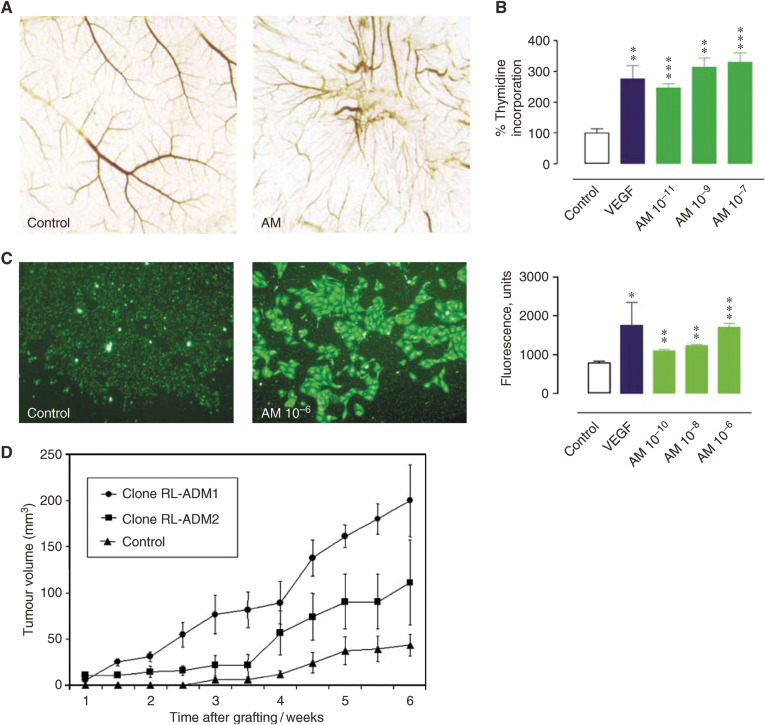
Angiogenic effects of adrenomedullin *in vitro* and *in vivo*. Adrenomedullin promotes (**A**) growth of new blood vessels *in vivo* in chicken chorioallantoic membrane assay (CAM), (**B**) proliferation of human endometrial microvascular endothelial cells and (**C**) migration of human dermal microvascular endothelial cells *in vitro*. CAM and proliferation assays were performed as described (from Zhao *et al*, 1998; Nikitenko *et al*, 2000). For migration assay, cells were seeded into the upper chamber of the transwell apparatus, and AM added into the lower chamber. The number of cells that migrated to the lower surface was dose-dependent (AM 10^–10^–10^–6^ M) compared to the control. (**D**) Adrenomedullin promotes formation of xenografted endometrial tumours. The suggested mechanisms include stimulation of autocrine growth and angiogenesis in AM-overexpressing RL95.2 tumours (Clones RL-ADM1 and RL-ADM2) (from Oehler *et al*, 2002). (**A** and **D**) are reproduced by kind permission of *Oncogene* (Nature Publishing Group) from Zhao *et al* (1998) and Oehler *et al* (2002). Each point represents the mean±s.e.m. (^*^P<0.05; ^**^P<0.01; ^***^P<0.001; as compared to controls).

A role for AM in physiological and pathological angiogenesis has been demonstrated using several *in vitro*, knockout mice and xenografted tumour models (Zhao *et al*, 1998; Hague *et al*, 2000; Nikitenko *et al*, 2000; Oehler *et al*, 2001; Fujita *et al*, 2002; Martinez *et al*, 2002; Kim *et al*, 2003) (Figure 2).

AM plays a role in the regulation of angiogenesis in the female reproductive tract (Nikitenko *et al*, 2000), during embryonic vascular development (Caron and Smithies, 2001; Shindo *et al*, 2001), and during vascular remodelling in response to ischaemia (Iimuro *et al*, 2004; Matsui *et al*, 2004). Thus, AM induces growth of human endometrial microvascular endothelial cells (Nikitenko *et al*, 2000). Studies using AM knockout mice suggest that AM is essential for vascular morphogenesis (Caron and Smithies, 2001; Shindo *et al*, 2001). Adrenomedullin also augments blood flow recovery and collateral capillary development in response to acute ischaemia (Abe *et al*, 2003).

Similarly, AM is considered to be angiogenic in tumours. For example, in leiomyomas, benign myometrial tumours, AM expression correlates with vascular density and the endothelial cell proliferation index (Hague *et al*, 2000). In xenografted tumour models utilising human endometrial, breast or pancreatic tumour cell lines, vascular density or directed growth of blood vessels was increased in AM-overexpressing transfectants (Oehler *et al*, 2001; Martinez *et al*, 2002; Ishikawa *et al*, 2003). Similar effects were observed after xenografting human glioblastoma cells, that were known to express high levels of endogenous AM (Ouafik *et al*, 2002). These observations have been confirmed in AM-heterozygous knockout mice, where decreased xenografted tumour growth and reduced neovascularisation in ischaemic conditions were observed (Iimuro *et al*, 2004).

A role for AM in vascular maturation has also been suggested (Iwase *et al*, 2005). AM stimulation of vascular smooth muscle cell (VSMC) migration and endothelium-independent vasodilatation may contribute to vascular maturation. Recent studies show that AM not only enhances the differentiation of bone marrow-derived mononuclear cells into endothelial cells but also facilitates formation of mature vessels that include VSMC (Iwase *et al*, 2005). In contrast, others demonstrated that endogenous AM could protect against pulmonary vascular remodelling induced by hypoxia or limit the arterial intimal hyperplasia induced by injury (Kawai *et al*, 2004; Matsui *et al*, 2004).

Finally, a role for PAMP in angiogenesis has also been suggested (Martinez *et al*, 2004b).

### Adrenomedullin and apoptosis

Adrenomedullin inhibits apoptosis of both endothelial and tumour cells. For example, AM suppresses serum deprivation-induced apoptosis in rat endothelial cells (Kato *et al*, 1997). Stably transfected AM-overexpressing endometrial tumour cells show resistance to hypoxia-induced apoptosis via a bcl-2-mediated mechanism (Oehler *et al*, 2002). Furthermore, AM-overexpressing breast carcinoma cells show lower levels of proapoptotic factors such as Bax, Bid and caspase 8, concomitant with higher resistance to apoptosis (after serum deprivation), than cells transfected with the empty vector (Martinez *et al*, 2002).

## ADRENOMEDULLIN RECEPTORS AND SIGNALLING IN TUMOUR BIOLOGY

Currently CL is considered to be the main receptor mediating AM effects (McLatchie *et al*, 1998; Poyner *et al*, 2002). In normal tissues CL is predominantly expressed in microvascular endothelial cells, supporting the view that this GPCR is potentially a major regulator of the effects of AM on vasculature (Nikitenko *et al*, 2003). Calcitonin-receptor-like receptor gene transcription is upregulated in endothelial cells in hypoxia. Simultaneous transcriptional upregulation of CL and it ligand AM in endothelial cells might play a significant role in vascular responses to hypoxia and ischaemia by creating a survival loop. Calcitonin-receptor-like receptor is also upregulated in some human tumours (Nikitenko *et al*, 2003).

In vascular endothelial cells, activation of phosphatydylinositol 3′kinase (PI3K/Akt), mitogen-activated protein kinase (MAPK) and focal adhesion kinase (p125FAK) plays a role in AM-induced angiogenesis (Figure 1). Thus, AM-stimulated migration of human umbilical vein endothelial cells (HUVEC) is inhibited by PI3K- or MAPK-inhibitors (Kim *et al*, 2003). Nitric oxide (NO) upregulation by AM has been implicated in endothelium-dependent vasodilatation (reviewed in Hinson *et al*, 2000). Adrenomedullin reduces endothelial apoptosis via a c-AMP-independent mechanism, and by upregulation of transcription factor Max (Kato *et al*, 1997; Shichiri *et al*, 1999).

The mitogenic activity of AM on VSMC is mediated via a cAMP-dependent pathway and/or a MAPK (reviewed by Hinson *et al*, 2000). Endothelium-independent vasodilatory effects of AM are mediated via a cAMP-dependent mechanism in VSMC (Figure 1). Adrenomedullin-stimulated VSMC migration is inhibited by wortmannin, a PI3K inhibitor, and it has been suggested that activation of PI3K/Akt by the peptide *in vivo* contributes to recruitment of VSMC to a newly formed capillary network (Iwase *et al*, 2005). Stimulation of vascular maturation by a direct effect of AM on VSMC is in sharp contrast to VEGF that stimulates new vessel formation but not maturation. Whether AM and VEGF act synergistically or independently remains to be elucidated. Two recent studies have shown that AM enhances vascular endothelial growth factor (VEGF)-induced capillary formation by HUVEC *in vitro*, but there is disagreement on the ability of AM to stimulate VEGF production in these cells (Fernandez-Sauze *et al*, 2004; Iimuro *et al*, 2004).

Transfected tumour cells overexpressing AM have higher levels of oncogenic proteins such as Ras, Raf, PKC and MAPKp49 and incorporate more bromodeoxiuridine (after serum deprivation) compared to controls (Martinez *et al*, 2002) (Figure 1). Adrenomedullin-mediated upregulation of antiapoptotic factors in hypoxia or downregulation of pro-apoptotic factors contributes to tumour cell survival (Oehler *et al*, 2001; Martinez *et al*, 2002). Further, AMBP-1 enhances AM-mediated growth in breast cancer cell lines and has been recently implicated in a mechanism leading to tumour cell survival by resistance to complement-mediated lysis (reviewed in Zudaire *et al*, 2003). However, the role of AMBP-1 in protection of tumour cells from apoptosis is unknown and further studies are needed.

## ADRENOMEDULLIN AND TUMOUR THERAPY

Several strategies have been proposed to inhibit AM-induced angiogenesis and tumour cell growth and survival. Among these are approaches that aim to: (1) modulate AM expression (AM mRNA rybozyme), (2) alter AM binding to its receptor (anti-AM blocking antibodies, receptor antagonists, truncated peptides, e.g., AM_22–52_ and CGRP_8–37_, and small nonpeptide molecules), or (3) inhibit specific AM-induced intracellular signalling pathways (e.g. using anti-CL or anti-RAMPs blocking antibodies, inhibitors of PI3K/Akt or MAPK pathways and other angiostatic molecules, e.g., vinblastine) (Table 1). Several such strategies have successfully attenuated AM-induced signalling and inhibited endothelial cell growth and migration, or increased tumour cell apoptosis. Studies in knockout mice have demonstrated that loss of AM results in elevated blood pressure (Shindo *et al*, 2001), which could be a problem in blocking therapies.

### CLINICAL RELEVANCE OF ADRENOMEDULLIN AND ITS RECEPTORS IN CANCER AND FUTURE TRANSLATIONAL RESEARCH

Although the role of AM has been studied using *in vitro* and murine xenografted tumour models, studies in man have been limited to use of cancer cell lines and a few tumours.

Currently, immunohistochemistry and RT–PCR have been the first choice with which to study AM expression and distribution in human tumour tissues, but there is a paucity of quantitative data. There is also little data concerning which cells express the messenger RNA within tumours (Rocchi *et al*, 2001). A few studies have been performed to measure peptide levels in blood or tumour tissues from sufficient number of patients to enable statistical analysis (Satoh *et al*, 1995; Oehler *et al*, 2003). For example, it has been shown that the plasma AM level in breast cancer patients correlates with primary tumour size and lymph node metastasis (*P*=0.002), as measured by radioimmunoassay (Oehler *et al*, 2003). However, despite the fact that plasma AM may be an independent predictor of lymph node metastasis, it was concluded that it is unlikely to be a useful tumour marker for the detection of breast cancer.

The correlation of AM mRNA expression with tumour stage has been demonstrated only for human glioblastomas (Ouafik *et al*, 2002). Its correlation with vascular density has been demonstrated in renal tumours and uterine leiomyoma (Hague *et al*, 2000; Fujita *et al*, 2002). However, the small patient number used in the first two studies (with a maximum of six and 12 samples per group, respectively) limits reliable conclusions. Studies by Hague *et al* (2000) did not examine correlations within leiomyoma-bearing uteri, making it difficult to assess the potential of anti-AM strategies and their possible effects on surrounding myometrium.

Finally, no studies have yet used quantitative methods to assess the mRNA and protein expression levels for components of the AM receptor system (CL and RAMPs). Information on the distribution, regulation and function of endogenous AM receptors is poor, and data on their role in human tumour biology is not available. CL mRNA might be upregulated in some tumour types (Nikitenko *et al*, 2003), but little information is available on protein expression, receptor distribution, or concerning CL-mediated signalling pathways involved in human tumours. RAMPs 2 and 3 mRNAs have also been found in some tumour types, but no studies have characterised the expression of functional endogenous AM receptors and their role in tumorigenesis. Thus there is still much to be learned about their role in human cancer especially in regards to translational research (Liang, 2003).

## CONCLUDING REMARKS, UNANSWERED QUESTIONS AND FUTURE DIRECTIONS

### Validation of adrenomedullin as a tumour expressed gene

Several studies have shown that expression of AM by tumour models enhances tumour growth and angiogenesis, whereas blocking AM antibodies have the reverse effect. While these observations point to a potential role for AM and its receptors in tumour angiogenesis, their role in human cancer is as yet poorly defined. There exists an urgent need for a greater assessment of AM as a novel tumour marker. In the first place this requires a knowledge of the expression at the mRNA and protein level of AM and CL in human normal and tumour tissues. Ideally, this would involve correlation of expression levels in patients with clinico-pathological data. Expression studies have shown AM (like VEGF) to be widely expressed in normal adult tissue. Expression is greatest in the placenta and endometrium that are undergoing active angiogenesis, but was also significant in the adrenal medulla, lung and kidney (Kitamura *et al*, 1993b). A more detailed analysis nevertheless suggests that in some cases tumour expression is greater than that in corresponding normal tissue, for example, kidney where it is many fold higher (L. Nikitenko, unpublished work). Several *in vitro* studies have demonstrated that hypoxia upregulates AM expression in tumour cells and this needs to be validated *in vivo* by, for example, *in situ* hybridisation (Rocchi *et al*, 2001). The accurate determination of AM peptide in tissues is far from straightforward. There is no readily available ELISA for AM leaving radioimmunoassay as the most sensitive assay (Satoh *et al*, 1995; Oehler *et al*, 2003). Interpretation of the results is confounded by the existence of adrenomedullin binding protein-1 (AMBP-1) and the question of how much AM is bound to AMBP-1 or free (reviewed in Zudaire *et al*, 2003).

### Adrenomedullin as a therapeutic target

There is a need to identify the tumour types where expression of AM and its receptors correlate with, for example, vascularisation, prognosis and resistance to chemotherapy. This would single out tumours in which AM plays a role and where anti-AM therapeutic intervention may have potential. That antibodies to AM reduce tumour growth in animal models was a significant observation reminiscent of similar observations with anti-VEGF antibodies a decade earlier. The VEGF studies eventually led to the development of the anti-VEGF drug ‘Avastin’ and its efficacy in the treatment of advanced metastatic colon cancer (Hurwitz *et al*, 2004). Nevertheless, Avastin-treated tumours eventually re-grow despite continued treatment, and it is potent angiogenic factors such as AM that are likely to be responsible for the breakthrough angiogenesis. One consideration is potential side effects of AM-blocking antibody, since loss of AM results in elevated blood pressure as demonstrated by studies in AM knockout mice (Shindo *et al*, 2001). A detailed knowledge of the interaction of AM with its receptors, and mechanisms of their expression and function will provide insights that are essential for the future development of chemical compounds and antibodies that can modulate the activity of endogenous AM receptors. Such drugs may have potential for antitumour therapy.

## Figures and Tables

**Table 1:** Specific and nonspecific modulators of adrenomedullin-induced effects

**Target**	**Drug**	**Mode of action/biological activity**	**Side-effects/disadvantages**	**Antiangiogenic potential**	**Reference**
*Specific modulators of AM-induced effects*					
Ligand (adrenomedullin)	AM mRNA rybozyme	AM mRNA degradation	Lack of reliable targeted delivery	Not tested	Taylor and Samson (2002)

	AMBP-1 (binding protein)	Affinity to receptor is unaltered Protection of peptide from degradation by proteases	Not tested	Not tested	Reviewed by Zudaire *et al* (2003)

	Anti-AM blocking antibody	Inhibition of AM activity	Not tested	Decreased mean vessel area in tumour xenografts	Ouafik *et al* (2002)

	Positive nonpeptidic regulators	Binding to AM	Vasodilation *in vivo*	Not tested	Martinez *et al* (2004a), (2004b)
	Negative nonpeptidic regulators	Binding to AM	Vasoconstriction	Not tested	Martinez *et al* (2004a), (2004b)

Receptor	Fragmentary peptides				
	AM22-52 (receptor antagonist) CGRP8-37 (receptor antagonist)	Competition with ligand for binding to the receptor	Short half-life Short half-life	Not tested Not tested	Poyner *et al* (2002) Poyner *et al* (2002)

	Anti-CL blocking antibody Anti-RAMPs blocking antibodies	Direct interaction with receptor has been suggested	Not tested Not tested	Inhibit HUVEC migration and capillary tube formation *in vitro*	Fernandez-Sauze *et al* (2004)

*Nonspecific modulators of AM-induced effects*					
Signalling cascades (secondary messengers)	Wortmannin (PI3K inhibitor) PD98059 (MAPK inhibitors)	Inhibition of ligand-induced phospohorylation of secondary messengers/kinases	Nonspecific Nonspecific	Prevent angiogenesis *in vitro* and *in vivo*	Kim *et al* (2003) Kim *et al* (2003)

Unknown	Vinblastine	Interaction with cytoskeleton	Nonspecific	Interruption of AM-induced capillary-like tube formation	Ribatti *et al* (2003)
